# An mTOR inhibitor discovery system using drug-sensitized yeast

**DOI:** 10.1007/s11357-025-01534-8

**Published:** 2025-01-30

**Authors:** Anna K. Breen, Sarah Thomas, David Beckett, Matthew Agsalud, Graham Gingras, Judd Williams, Brian M. Wasko

**Affiliations:** Department of Biomedical Sciences, Western University of Health Sciences, Lebanon, OR 97355 USA

**Keywords:** mTOR, Rapamycin, Yeast

## Abstract

Inhibition of the target of rapamycin (TOR/mTOR) protein kinase by the drug rapamycin extends lifespan and health span across diverse species. However, rapamycin has potential off-target and side effects that warrant the discovery of additional TOR inhibitors. TOR was initially discovered in *Saccharomyces cerevisiae* (yeast) which contains two TOR paralogs, *TOR1* and *TOR2*. Yeast lacking functional Tor1 are viable but are hypersensitive to growth inhibition by TORC1 inhibitors, which is a property of yeast that can be exploited to identify TOR inhibitors. Additionally, yeast lacking FK506-sensitive proline rotamase (*FPR1*) or containing a *tor1-1* allele (a mutation in the Fpr1-rapamycin binding domain of Tor1) are robustly and selectively resistant to rapamycin and analogs that allosterically inhibit TOR activity via an *FPR1*-dependent mechanism. To facilitate the identification of TOR inhibitors, we generated a panel of yeast strains with mutations in TOR pathway genes combined with the removal of 12 additional genes involved in drug efflux. This creates a drug-sensitive strain background that can sensitively and effectively identify TOR inhibitors. In a wild-type yeast strain background, 25 µM of Torin1 and 100 µM of GSK2126458 (omipalisib) are necessary to observe *TOR1*-dependent growth inhibition by these known TOR inhibitors. In contrast, 100 nM Torin1 and 500 nM GSK2126458 (omipalisib) are sufficient to identify *TOR1-*dependent growth inhibition in the drug-sensitized background. This represents a 200-fold and 250-fold increase in detection sensitivity for Torin1 and GSK2126458, respectively. Additionally, for the TOR inhibitor AZD8055, the drug-sensitive system resolves that the compound results in *TOR1-*dependent growth sensitivity at 100 µM, whereas no growth inhibition is observed in a wild-type yeast strain background. Our platform also identifies the caffeine analog aminophylline as a *TOR1*-dependent growth inhibitor via selective *tor1* growth sensitivity. We also tested nebivolol, isoliquiritigenin, canagliflozin, withaferin A, ganoderic acid A, and taurine and found no evidence for TOR inhibition using our yeast growth-based model. Our results demonstrate that this system is highly effective at identifying compounds that inhibit the TOR pathway. It offers a rapid, cost-efficient, and sensitive tool for drug discovery, with the potential to expedite the identification of new TOR inhibitors that could serve as geroprotective and/or anti-cancer agents.

**Supplementary Information:**

The online version contains supplementary material available at 10.1007/s11357-025-01534-8.

## Introduction

The serine/threonine protein kinase known as the target of rapamycin (TOR or mechanistic/mammalian mTOR) was originally named for its discovery in *Saccharomyces cerevisiae* (yeast), where mutations in the two yeast TOR genes were found to suppress rapamycin-mediated inhibition of growth [19, 52]. mTOR is a highly conserved protein kinase that is a master regulator of cell growth and proliferation that responds to multiple external stimuli.

Genetic reduction of the mTOR signaling pathway extends the lifespan of *Caenorhabditis elegans* [61], *Drosophila melanogaste*r [25], yeast [23, 49], and mice [64]. Pharmacological inhibition of TOR by rapamycin extends lifespan in yeast [49], flies [6], and mice [18]. Interestingly, even a short duration of rapamycin treatment in middle-aged mice is sufficient to extend longevity [5].

The mTOR kinase functions within two distinct protein complexes, TORC1 and TORC2. TORC1 is a nutrient-responsive kinase that promotes anabolic (e.g., protein synthesis) and regulates catabolic (e.g., autophagy) processes to promote cell growth in response to appropriate nutrients [35]. TORC2 is less well studied and has distinct downstream effects [16]. Given that mTOR is a master regulator of cell growth and cell division, rapamycin and other TOR inhibitors are also of interest for cancer treatment [47] and prevention [7].

Rapamycin and its analogs (rapalogs) show promise as geroprotective and anti-cancer therapeutics, but multiple limitations may temper their application. One common concern is that rapamycin was first FDA-approved for the prophylaxis of organ transplant rejection, and thus, its immunosuppressive properties may increase susceptibility to infections. Despite this concern, some accumulating evidence suggests that rapamycin may have immunomodulatory benefits in the aged population. A meta-analysis of mouse studies concluded that treatment with rapamycin significantly increases the short-term survival of mice in response to acute pathogen infection [45]. In addition, a recent systematic review of human studies supports that rapamycin or rapalog treatment can promote beneficial effects for the aging immune system [30]. In a survey study of off-label use of rapamycin (consisting primarily of once weekly dosing), there was a statistically significant increase in the incidence of mouth ulcers reported by rapamycin users compared to a control group, as well as a non-statistically significant trend toward higher infection rates [24]. These studies highlight that the FDA approval of rapamycin as an immunosuppressant is not sufficient to dismiss its use as a geroprotective agent, though concerns persist.

Another concern is that chronic rapamycin treatment can result in metabolic disturbances due to off-target effects. This off-target effect is attributed to rapamycin binding free mTOR and preventing its assembly into TORC2 [53], thereby reducing TORC2 activity and impairing glucose tolerance (the ability to regulate blood sugar levels after a glucose load) in mice [27]. Modifying the treatment regimen of rapamycin can mitigate these adverse effects in animal models, improving both glucose regulation and immune function [2]. In humans, intermittent dosing similarly reduces rapamycin-induced glucose intolerance, although it does not fully eliminate it [3].

In order to accelerate the identification of novel mTOR inhibitors, the development of cost-effective and rapid assays is warranted. Ideal assays should be sensitive, specific, and amenable to high-throughput screening of molecular libraries. Developing novel processes for identifying TOR inhibitors can expedite drug discovery and reveal promising candidates for further investigation as potential geroprotective agents. Systems that can evaluate high-throughput combinations of compounds will also be useful.

**Fig. 1:**
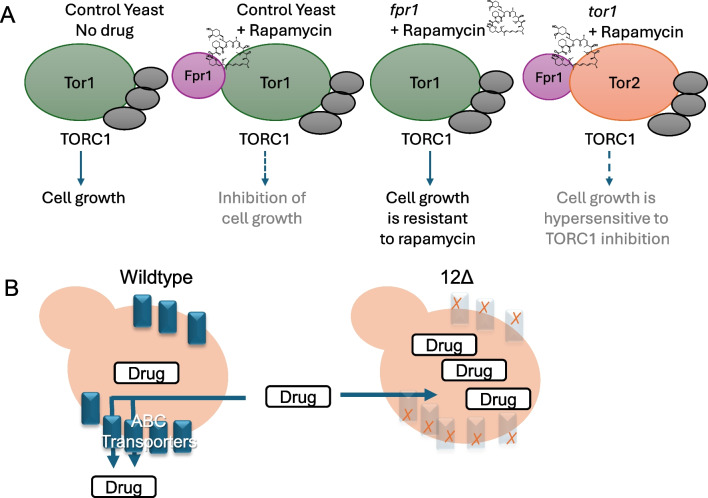
Representation of yeast strains used in this study. **A** Growth inhibition by rapamycin and rapalogs depends on Fpr1 for the allosteric inhibition of TOR complex 1 (TORC1). Wild-type yeast are sensitive to rapamycin-mediated growth inhibition, while *fpr1*-deficient mutant yeast exhibit robust resistance to rapamycin and rapalogs due to the requirement of Fpr1 for the allosteric drug mechanism involving binding with rapamycin and the Tor protein. In contrast, *tor1*-deficient mutant yeast are hypersensitive to rapamycin and other TORC1 inhibitors. The viability of yeast *tor1* mutants and the hypersensitivity to TORC1 inhibitors arises due to low residual activity of the yeast Tor2 protein functioning within TORC1, which remains susceptible to inhibition. Gray circles represent other protein components of the TORC1 complex. **B** Schematic comparing the wild-type strain background (BY4742 or BY4741) to the drug-sensitized 12Δ strain, which lacks multiple ABC transporters that can function as drug efflux pumps

*S. cerevisiae* contains two TOR genes, *TOR1* and *TOR2* that encode proteins which primarily function within the TORC1 and TORC2 complexes, respectively. Yeast lacking *TOR1* are viable but are hypersensitive to TORC1 inhibitors such as rapamycin due to a low residual activity of the Tor2 protein within the TORC1 complex (Fig. 1A). This property of yeast can be exploited to identify TORC1 inhibitors by assessing for compounds that selectively inhibit the growth of a yeast strain lacking a functional Tor1 protein [31]. Compounds that do not inhibit TORC1 activity are expected to similarly inhibit yeast growth in both Tor1-deficient and control strains or to have no growth inhibitory properties at all.

Additionally, yeast lacking functional Fpr1 or containing a *tor1-1* allele (an S1972R mutation in the Fpr1 binding domain of Tor1) are robustly and selectively resistant to rapamycin. This resistance arises because rapamycin allosterically inhibits TORC1 via an Fpr1 (FKBP12)-dependent mechanism [19]. Of note, the interaction of Fpr1 with TORC1 is primarily a drug-dependent phenomenon, and Fpr1 has no known endogenous role in TORC1 regulation in the absence of rapamycin.

As a unicellular organism evolved to survive in complex and harsh environments, yeast possess robust defensive mechanisms that help afford protection from their surroundings. These systems include numerous ATP-binding cassette (ABC) transport pumps capable of exporting potentially harmful substances including many drugs. Chinen et al. have developed a strain of yeast lacking 12 genes involved in drug efflux, which display increased sensitivity to a variety of compounds [10]. Using the 12geneΔhsr strain (referred to as 12Δ within this manuscript, Fig. 1B), the TOR pathway was modified to create a drug-sensitive system that can identify agents that inhibit yeast growth in a TORC1-dependent manner.

We sought to generate a drug-sensitized yeast model for use in the identification of TOR inhibitors. We hypothesized that using a drug efflux-deficient strain containing TOR pathway-associated mutations would allow for a system that will maintain selectivity while increasing sensitivity to growth inhibition via TOR inhibitors. Using multiple compounds known to inhibit the TOR pathway, we provide a proof of principle that the developed system can functionally be used to increase sensitivity for the identification of TOR inhibitors. We also tested a panel of molecules previously implicated to impact mTOR and/or lifespan, including nebivolol, isoliquiritigenin, α-lipoic acid, canagliflozin, withaferin A, ganoderic acid A, and taurine, and found no supportive evidence for TOR inhibition using our yeast growth-based assay.

## Results

Rapamycin and its analogs result in hypersensitivity with *tor1* mutants, while *fpr1* and *tor1-1* mutants exhibit robust resistance.

**Fig. 2:**
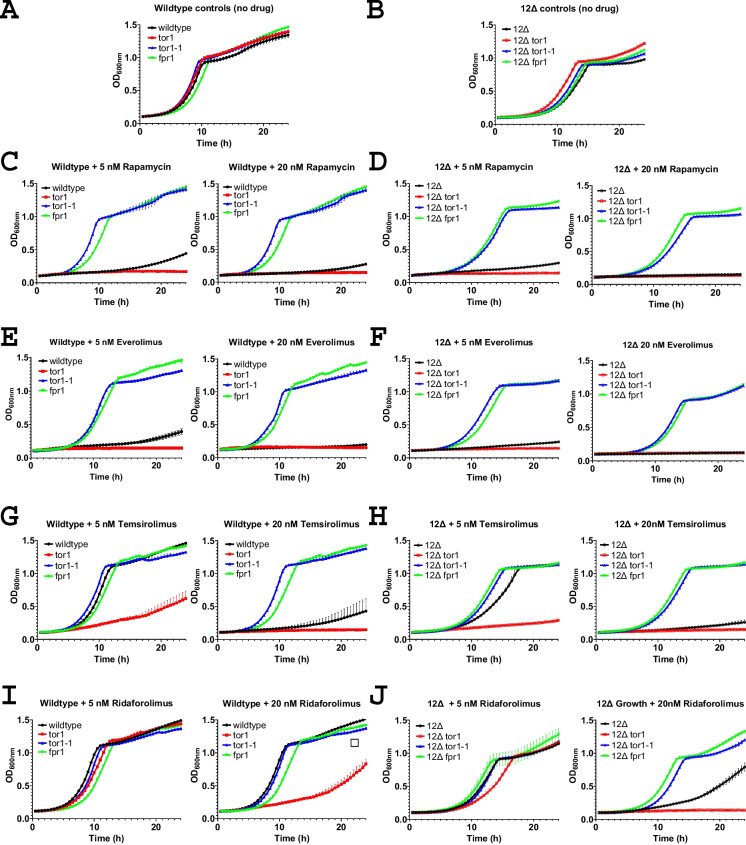
Rapamycin and its analogs result in hypersensitivity in *tor1*-deficient mutants, while *fpr1* and *tor1-1* mutants exhibit robust resistance. Growth curves are shown for **A** wild-type BY4742 haploids without drug, **B** 12Δ yeast without drug, **C** wildtype treated with 5 nM or 20 nM rapamycin, **D** 12Δ treated with 5 nM or 20 nM rapamycin, **E** wildtype treated with 5 nM or 20 nM everolimus, **F** 12Δ treated with 5 nM or 20 nM everolimus, **G** wild-type BY4742 treated with 5 nM or 20 nM temsirolimus, **H** 12Δ treated with 5 nM or 20 nM temsirolimus, **I** wild-type BY4742 treated with 5 nM or 20 nM ridaforolimus, and **J** 12Δ treated with 5 nM or 20 nM ridaforolimus. OD600 is the optical density measured at 600 nM. Closed shapes indicate wild-type strains and open shapes represent 12Δ strains. Black circles are control strains, red squares are *tor1* disruption mutant strains, blue triangles are *tor1-1* (Tor1-S1972R) mutants, and green triangles are *fpr1* disruption mutants. Error bars represent the standard deviation

A *tor1* strain is hypersensitive to rapamycin and the analogs ridaforolimus, temsirolimus, and everolimus, while *fpr1* or *tor1-1* mutant strains are strongly resistant [19, 31]. We set out to determine if the same profile of growth is retained in the 12Δ drug-sensitive genetic background. Growth curves for strains in the absence of drugs are shown for wild-type (Fig. 2A) and 12Δ yeast strains (Fig. 2B). Rapamycin strongly inhibited growth of wild-type (Fig. 2C) and the 12Δ strain (Fig. 2D) at 5 and 20 nM, and the *tor1-*deficient strains displayed an increased sensitivity to rapamycin at these concentrations, while the *fpr1* and *tor1-1* strains exhibited robust resistance to rapamycin.

Everolimus strongly inhibited the growth of wild-type (Fig. 2E) and the 12Δ strain (Fig. 2F) at 5 and 20 nM, and the *tor1-*deficient strains also displayed an increased sensitivity to everolimus at these concentrations, while the *fpr1* and *tor1-1* strains exhibited strong resistance to everolimus.

Temsirolimus was less potent compared to rapamycin and everolimus at 5 nM in both wild-type (Fig. 2G) and 12Δ backgrounds (Fig. 2H), while an increased sensitivity to growth inhibition was observable in the *tor1-*deficient strains. The *fpr1* and *tor1-1* strains exhibited resistance to growth inhibition in both the wild-type and 12Δ backgrounds.

Ridaforolimus appeared less potent than rapamycin and other rapalogs at inhibiting the growth of control strains in both wild-type (Fig. 2I) and 12Δ backgrounds (Fig. 2J), with an increased sensitivity observed in the 12Δ compared to the control background at 20 nM. The *tor1* strain exhibited enhanced sensitivity compared to the control strains in both the wild-type and 12Δ backgrounds at 20 nM ridaforolimus. The *fpr1* and *tor1-1* strains exhibited marked resistance to ridaforolimus in both wildtype and 12Δ.

The 12Δ strain background strongly increases sensitivity to ATP-competitive TOR inhibitors: AZD8055, Torin1, and GSK2126458 (omipalisib).

**Fig. 3:**
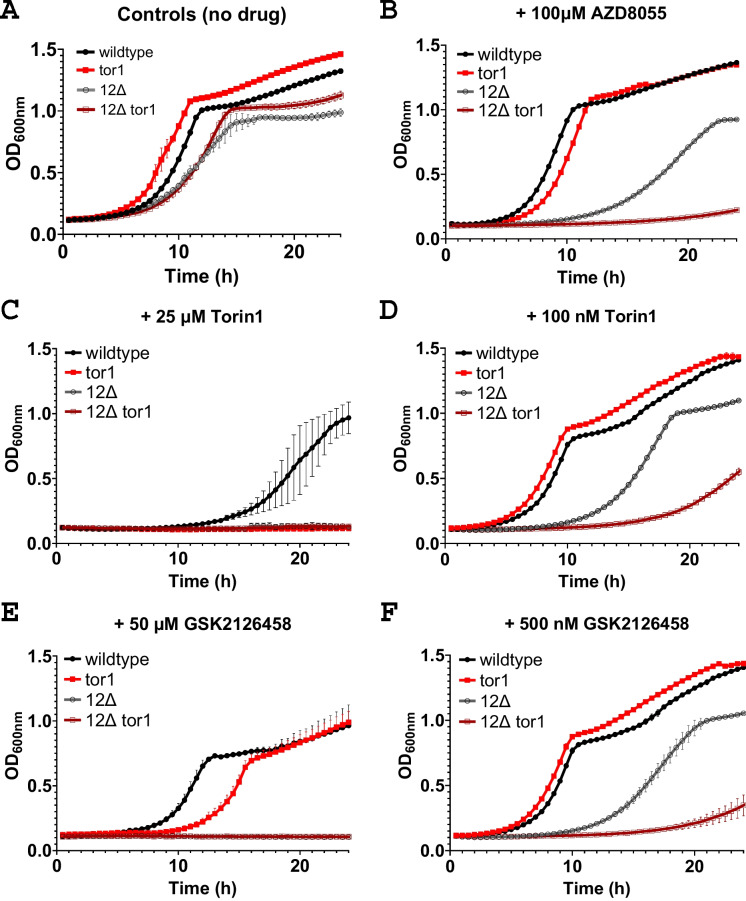
The ATP-competitive TOR inhibitors AZD8055, Torin1, and GSK2126458 (omipalisib) are more potently identifiable as *tor1*-dependent growth inhibitors in the 12Δ background. Growth curves on YPD media for wild-type BY4742 and 12Δ treated with **A** no drug, **B** 100 µM AZD8055, **C** 25 µM Torin1, **D** 100 nM Torin1, **E** 50 µM GSK2126458 (omipalisib), and **F** 500 nM GSK2126458 (omipalisib). OD600 is the optical density measured at 600 nM. Filled shapes are wild-type BY4742 strains, and open shapes are 12Δ strains. Black circles represent control strains, and red squares represent *tor1* disruption mutants. Error bars represent the standard deviation

ATP-competitive inhibitors constitute another class of mTOR inhibitors distinct from rapamycin and rapalogs. AZD8055 [11] and Torin1 [36] are ATP-competitive TORC1/TORC2 inhibitors, as is GSK2126458 (omipalisib), which additionally inhibits phosphoinositide 3-kinase (PI3K) [26]. Growth curves for no drug controls are indicated in Fig. 3A, and the compound AZD8055 did not inhibit growth of the control or *tor1* strain in the wild-type background at 100 µM (Fig. 3B). In the 12Δ yeast, 100 µM of AZD8055 inhibited growth of the control strain, and preferential inhibition in the *tor1* strain was observed. At 25 µM, Torin1 had a growth inhibitory effect in the control wild-type strain, and a selective inhibition of the *tor1* strain was observed, while no growth of either the control or *tor1* strain was observed at 25 µM torin1 for the 12Δ strains (Fig. 3C). Treatment with 100 nM Torin1 resulted in no growth inhibition observed in the wild-type strain background, but the 12Δ control strain was inhibited, and a selective inhibition of the 12Δ *tor1* strain was observable (Fig. 3D). Treatment with 50 µM GSK2126458 resulted in a preferential inhibition of the *tor1* strain compared to the control strain in the wild-type background, and no growth was observed in the 12Δ control or *tor1* strains (Fig. 3E). At 500 nM, GSK2126458 did not inhibit growth of the wild-type background strains, but inhibited the growth of the 12Δ strains, with a preferential inhibition of the 12Δ *tor1* strain observed (Fig. 3F). All of the tested ATP-competitive inhibitors were identified as having *tor1*-dependent sensitivities using the drug-sensitized system, while a wild-type system was unable to identify one even at high concentration. The two others were detected as having *tor1*-sensitive growth at ~ 200-fold lower concentrations in the 12Δ strain.

The 12Δ strain displays increased growth sensitivity to caffeine, and its analog aminophylline and *tor1* mutants are selectively sensitive to growth inhibition.

**Fig. 4:**
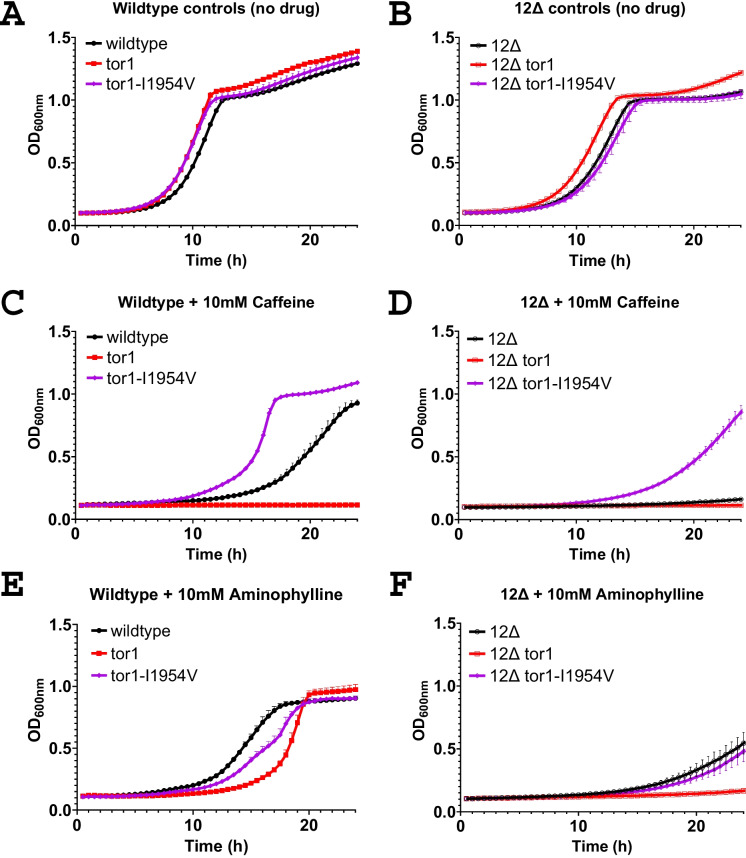
The 12Δ strain displays increased growth sensitivity to caffeine and its analog aminophylline, and *tor1* mutants are selectively sensitive to growth inhibition. Growth curves on YPD media for **A** wild-type control BY4742, *tor1*, and *tor1-2* (Tor1-I1954V) without drug; **B** 12Δ control, *tor1*, and *tor1-2* (Tor1-I1954V) without drug; **C** wild-type BY4742, *tor1*, and *tor1-2* (Tor1-I1954V) with 10 mM caffeine; **D** 12Δ control, *tor1*, and *tor1-2* (Tor1-I1954V) with 10 mM caffeine; **E** wild-type control BY4742, *tor1*, and Tor1-I1954V with 10 mM aminophylline; and **F** 12Δ control, *tor1*, and Tor1-I1954V with 10 mM aminophylline. OD600 is the optical density measured at 600 nM. Filled shapes are wild-type BY4742 strains, and open shapes are 12Δ strains. Black circle shapes are control strains, red squares are *tor1* strains, and purple diamonds are tor1-I1954V mutants. Error bars represent the standard deviation

Caffeine has previously been found to inhibit yeast growth in a *tor1*-dependent manner [51]. Controls without drug are indicated for the wild-type (Fig. 4A) and 12Δ strain backgrounds (Fig. 4B) with control, *tor1*, and an additional strain containing a *TOR1* allele encoding for an I1954V amino acid substitution previously identified as a caffeine-resistant mutation [51]. Caffeine at 10 mM inhibited growth of the control strain of a wild-type background, while the *tor1*-deficient mutant was preferentially inhibited, and the I1954V mutation conferred resistance (Fig. 4C). In the 12Δ background with 10 mM caffeine, an increased sensitivity of the 12Δ control strain was observed compared to the wild-type strain, and the *tor1*-deficient mutant also displayed a potent growth inhibition and the I1954V allele retained resistance (Fig. 4D). The analog of caffeine, aminophylline, at 10 mM displayed moderate preferential inhibition of growth of the *tor1*-deficient strain in the wild-type background; however, no resistance was noted when Tor1 contained the I1954V mutation (Fig. 4E). In the 12Δ background, 10 mM aminophylline inhibited growth of the control strain and Tor1-I1954V mutant strain, and an increased sensitivity of the *tor1*-deficient mutant was observable (Fig. 4F). No resistance was observed for caffeine or aminophylline in *fpr1* and *tor1-1* strains (data not shown).

We assessed a variety of additional compounds with potential TORC1 effects using our drug-sensitive model (Supplemental Table 1). We found that ganoderic acid A, α-lipoic acid, and taurine did not affect the growth of the 12Δ strain at the concentrations tested. Nebivolol, canagliflozin, isoliquiritigenin, and withaferin A were found to selectively inhibit growth in the 12Δ background; however, no observable selective sensitivity in the *tor1* strain was observed at the tested concentrations (Supplemental Table 1).

## Discussion

Rapamycin and rapalogs function as allosteric mTOR inhibitors that require binding with the protein FKBP12/Fpr1 and inhibit mTOR by binding to the Fkbp12 binding domain (FBD) within TOR. In the absence of Fpr1, or when a *tor1-1* (S1972R) mutation is present, yeast cells become insensitive to rapamycin up to ~ 100 µM [52]. With rapamycin and other rapalogs, the 12Δ background strains retained relatively similar growth profiles overall to the control ABC transporter proficient strain. This suggests that rapamycin and analogs are not likely to be efficiently effluxed from yeast cells, perhaps in part due to the tight binding with Fpr1, which may contribute to the relative potency of these molecules.

The 2nd generation mTOR inhibitors function as ATP-competitive inhibitors of TOR with variable specificity. AZD8055 [11] and Torin1 [36] are ATP-competitive TORC1/TORC2 inhibitors. GSK2126458 (omipalisib) is an ATP-competitive inhibitor of PI3K and mTORC1/mTORC2 [26]. The increased sensitivity of yeast lacking multiple plasma membrane ABC transporter pumps to these ATP-dependent mTOR inhibitors suggests that these compounds are normally actively effluxed from yeast cells. GSK2126458 at 10 µM is reported to extend the lifespan of *C. elegans* when treatment is started in adulthood [44], and it is also found to extend the lifespan of *C. elegans* by 35% by the ongoing Million Molecule Challenge [4], which uses a WormBot lifespan assay [46] for high-throughput longevity drug discovery. Of note, rapamycin effects on *C. elegans* longevity are potentially confounded by precipitation issues at the reported concentrations [38, 44]. Whether the GSK2126458 lifespan benefit is fully or partially dependent on a non-mTORC1 target such as PI3K may be of continued interest. Particularly of note, the first identified genetic longevity modifier in *C. elegans*, age-1, is a PI3K [15, 43]. Caffeine and its analogs also extend worm longevity and can dually inhibit mTOR and PI3K (discussed further below).

Caffeine is a widely consumed substance and a large body of literature surrounds the numerous biological effects. In yeast, we find that both caffeine and the analog aminophylline inhibit yeast growth in a *tor1*-dependent manner and the 12Δ strain is sensitized to both. Loss of *TOR1* has previously been found to sensitize yeast cells to caffeine [31, 51], and growth suppression and adaptation experiments using high concentrations of caffeine have identified mutations in *TOR1* that confer caffeine resistance [17, 42, 51]. Overexpression of the ABC transporters Snq2 and Pdr5 [59] and mutations in *PDR5* and *PDR1* have been found to increase yeast caffeine resistance [17, 42, 57], which supports our finding that the 12Δ strain, which lacks Pdr1, Pdr5, and Snq2, is sensitized to caffeine as well as the analog aminophylline. The Tor1-I1954V mutation previously identified to confer resistance to caffeine [51] also confers resistance to caffeine in the sensitized 12Δ background. This provides support that similar mechanisms of resistance are retained in this strain background, which helps to support the use of this genetic background for drug mechanism of action discovery [9]. Despite the structural similarity of aminophylline to caffeine, the Tor1-I1954V mutation did not confer similar resistance to aminophylline. This suggests that the methyl group present on caffeine at N7, which is absent from aminophylline, may bind Tor1 within the FRB region containing I1954.

Caffeine extends the lifespan of yeast [50, 62] and worms [8, 13, 32, 37, 58]. Li et al. find that the lifespan effect of caffeine is dependent on the IGF-1 pathway [13], which can act upstream of mTOR signaling [21]. It remains of interest whether caffeine’s impact on longevity in these model organisms is directly or indirectly dependent on mTOR. *S. cerevisiae* Tor1-I1954V confers resistance to caffeine-mediated growth inhibition [51], and *C. elegans* contain a valine at the equivalent position in *let-363*, suggesting that the *C. elegans* mTOR protein may be more resistant to caffeine compared to yeast and humans. Mutation of this residue in *C. elegans* to alanine could be used to determine if it reduces the concentration of caffeine necessary for lifespan extension, which could provide evidence in regard to the role of the TOR pathway in caffeine-mediated longevity in worms.

Aminophylline consists of the caffeine analog theophylline with ethylenediamine to improve solubility. Theophylline has been reported to extend worm lifespan [32]. Theophylline at 5 mM inhibits immunopurified mTOR in vitro and inhibits mTOR kinase activity in 3T3-L1 adipocyte cells [54]. Caffeine and theophylline are reported to inhibit mTOR as well as PI3K, with subunit p110δ most potently with an IC_50_ of 75 µM [14]. Aminophylline has also been reported to reduce the elevated phospho-S2448 mTOR in an in vivo rat model of chronic renal failure [34].

In mammals, the primary molecular mechanisms of caffeine include phosphodiesterase inhibition, antagonism of adenosine receptors, and alterations in intracellular calcium [12]. Caffeine’s effects on PI3K may also be physiologically relevant [14]. Effects of caffeine on mTOR have been observed primarily at high concentrations, so the physiological relevance of mTOR inhibition in mammalian cells is unclear. Multiple beneficial effects have been observed for coffee consumption and human age-associated diseases as well as a reduction of all-cause mortality,however, many beneficial effects have also been observed with decaffeinated coffee [60], suggesting caffeine-independent effects. This is further complicated as decaffeinated coffee still contains caffeine at lower concentrations [39].

We also tested a variety of additional compounds for potential impacts on the TOR pathway using our yeast drug-sensitive cell model. Canagliflozin has been found to impact the mTOR pathway [20] and to extend the lifespan of male mice [40, 41]. Nebivolol has been suggested to inhibit TORC1, but no lifespan extension was observed in mice under the conditions tested [41]. Alpha-lipoic acid may reduce mTOR signaling [33, 65]. Isoliquiritigenin and withaferin A were also tested based on their predicted potential as rapamycin mimetic molecules [1]. Ganoderic acid A was also tested as it is commercially available, and butylated ganoderic acid A was predicted to be an FKBP12-dependent mTOR inhibitor using a machine learning approach [48]. Taurine declines with age and supplementation can promote longevity in worms and mice, although the molecular mechanism is unclear [56]. At the concentrations tested, taurine, ganoderic acid A, and alpha-lipoic acid had no effect on the 12Δ yeast strains, suggesting that the compounds may not inhibit yeast TOR and/or were unable to efficiently enter into yeast cells. Canagliflozin, isoliquiritigenin, withaferin A, and nebivolol at the concentrations tested had growth inhibitory effects in the 12Δ strain,however, no observations of selective sensitivity of the *tor1* strain were observed. This suggests that the growth inhibitory activity of these compounds may be *TOR1*-independent. Canagliflozin, isoliquiritigenin, withaferin A, and nebivolol caused growth inhibition in 12Δ, but not the wild-type strain background, suggesting that they may be actively effluxed from yeast cells by ABC transporters that are absent in the 12Δ strain.

Using this yeast-based system for identifying *tor1*-dependent inhibitors requires an optimized concentration (i.e., range finding for optimal doses) for detection of the preferential inhibition of the *tor1*-deficient strain. However, identifying *fpr1*-dependent inhibition such as that observed with rapamycin is observable over a much larger concentration range and can be more easily qualitatively identified. In addition to rapamycin, tacrolimus (FK506) is another natural product from *Streptomyces hygroscopicus* that inhibits growth in a FKBP12-dependent manner, yet the downstream molecular target of the drug-FKBP12 complex, calcineurin, is distinct. Of note, WDB0002, CEP250, meridamycin, and antascomycins A–E are additional FKBP12-dependent natural products that lack identified downstream targets [52]. Considering the robust rapamycin and rapalog resistance of the yeast *fpr1* mutant and the fact that various natural products can inhibit specific downstream targets through Fpr1-dependent mechanisms, it may be worthwhile to search for additional natural products with Fpr1-dependent effects on yeast growth.

Given the high-throughput capability of this system, molecular libraries including natural product libraries could be assayed using this approach. Future use of this system could also include identifying the effects of mixtures of compounds including assessing for synergistic interactions using yeast growth. Additionally, disrupting cell wall integrity [10] could enhance the entry of compounds and further increase the sensitivity of the system.

## Methods

### Reagents

Reagents used in this study include rapamycin (LC Laboratories, Woburn, MA, USA), everolimus, temsirolimus, ridaforolimus, AZD8055, Torin1, GSK, canagliflozin hemihydrate, alpha-lipoic acid, nebivolol (APExBIO, Houston, TX, USA), caffeine, theophylline, taurine (Thermo Fisher Scientific, Ward Hill, MA, USA), withaferin A (Adipogen, San Diego, CA, USA), ganoderic acid A (MCE, Monmouth Junction, NJ, USA), isoliquiritigenin, and aminophylline (TCI, Tokyo, Japan).

### Yeast strains

Yeast strains used in this study are shown in Supplemental Table 2. CRISPR was used for yeast strain generation similarly to as previously described [28, 29, 55]. Briefly, sgRNA targeting sequences were selected using Geneious Prime software and were engineered to contain SmiI and BclI sites for cloning. Oligonucleotides (Azenta Genewiz, South Plainfield, NJ, USA) containing the sgRNA targeting sequence were hybridized to double-stranded DNA and then ligated using T4 DNA ligase into SmiI and BclI (NEB, Ipswich, MA, USA) digested pML104. Plasmids were confirmed to contain the desired sgRNA by PCR verification. Oligonucleotide sequences used in this study are shown in Supplemental Table 3. Yeast strains (either BY4742 or the 12Δ strain) were transformed using a EZ Yeast Transformation kit (Zymo Research Corporation, Irvine, CA, USA) with the indicated sgRNA targeting CRISPR plasmid along with a corresponding repair template. Sanger sequencing was performed to verify the presence of the desired mutations. The Saccharomyces Genome Database (SGD) was a critical resource used in support of this study [63].

### Yeast liquid culture growth assays

Overnight cultures were inoculated using a single colony and grown at 30 °C with shaking in YPD media consisting of 1% Bacto Yeast Extract (BD, Franklin Lakes, NJ, USA), 2% Bacto Peptone (BD, Franklin Lakes, NJ, USA), and 2% dextrose (Sunrise Science Products, Knoxville, TN, USA). The following day, the overnight culture was diluted ~ 1/100 into YPD, and all samples were adjusted to an equal initial optical density (OD) of 0.10 ± 0.02 using a Biochrom WPA Biowave CO8000 cell density meter. A 150 µL aliquot of the diluted overnight culture was added to 96 well plates, with the exception of blanks, which contained media only. To the samples, a 1.5 µL aliquot of the respective chemical compound was added to each treatment group with the exception of caffeine and aminophylline, of which 15 µL of the drug was added. DMSO vehicle controls were included where appropriate. Growth assays were performed using an Epoch2 Microplate Reader (BioTek, Winooski, VT, USA) with Gen6 software version 1.03.01. 96-well microplates were incubated at 30 °C with continuous double orbital shaking with a frequency of 807 cycles per minute (cpm). A lid temperature gradient was set to + 2 °C to prevent condensation, and absorbance at 600 nm (OD600) was measured every 30 min for 24 h. Of note, 384-well plates were also tested with settings as above except with 60 µL volume and 269 cpm (6 mm orbital) shaking based on a previous report [22], which was found to generate fermentative growth curves in the BY4742 strain backgrounds (data not shown). A standardized blank OD value was determined by taking the mean value of all blanks across all experiments and subtracting from all datasets. Dose–response curves were first performed to identify the concentrations used within this study. All individual experiments were performed using replicates across three wells which were averaged, and each experiment was repeated independently three or more times. Individual representative experimental data are shown.

## Supplementary Information

Below is the link to the electronic supplementary material.

Supplementary file1 (XLSX 9 KB)

Supplementary file2 (XLSX 63 KB)

Supplementary file3 (XLSX 24 KB)
